# Hyperglycemia-associated alterations in cellular signaling and dysregulated mitochondrial bioenergetics in human metabolic disorders

**DOI:** 10.1007/s00394-016-1212-2

**Published:** 2016-04-15

**Authors:** George B. Stefano, Sean Challenger, Richard M. Kream

**Affiliations:** MitoGenetics LLC, 3 Bioscience Park Drive, Suite 307, Farmingdale, NY 11735 USA

**Keywords:** Mitochondria, Glucose, Hyperglycemia, Diabetes, ATP, Aerobic glycolysis, Advanced glycation end-products, Receptor for advanced glycation end-products, Hexosamine biosynthetic pathway, Hexosamine biosynthetic pathway

## Abstract

**Purpose:**

The severity of untreated or refractory diabetes mellitus has been functionally linked to elevated concentrations of free plasma glucose, clinically defined as hyperglycemia. Operationally, the pathophysiological presentations of prolonged hyperglycemia may be categorized within insulin-dependent and insulin-independent, type 1 and type 2 diabetic phenotypes, respectively. Accordingly, major areas of empirical biomedical research have focused on the elucidation of underlying mechanisms driving key cellular signaling systems that are significantly altered in patients presenting with diabetes-associated chronic hyperglycemia.

**Methods:**

Presently, we provide a translationally oriented review of key studies evaluating the aberrant effects of hyperglycemia on two major signaling pathways linked to debilitating cellular and systemic effects via targeted disruption of mitochondrial bioenergetics: (1) advanced glycation end-products (AGEs)/and their cognate receptor for advanced glycation end-products (RAGEs), and (2) the hexosamine biosynthetic pathway (HBP).

**Results:**

In preclinical models, cultured vascular endothelial cells exposed to hyperglycemic glucose concentrations were observed to produce enhanced levels of reactive oxygen species (ROS) functionally linked to increased formation of AGEs and expression of their cognate RAGEs. Importantly, inhibitors of AGEs formation, mitochondrial complex II, or un-couplers of oxidative phosphorylation, were observed to significantly reduce the effects of hyperglycemia on ROS production and cellular damage, thereby establishing a critical linkage to multiple levels of mitochondrial functioning. Hyperglycemia-mediated enhancement of mitochondrial ROS/superoxide production in vascular endothelial cells has been functionally linked to the shunting of glucose into the HBP with resultant long-term activation of pro-inflammatory signaling processes. Additionally, exposure of cultured cells to hyperglycemic conditions resulted in enhanced HBP-mediated inhibition of protein subunits of mitochondrial respiratory complexes I, III, and IV, intimately associated with normative cellular bioenergetics and ATP production.

**Conclusions:**

Convergent lines of evidence link chronic hyperglycemic conditions to aberrant expression of AGEs/RAGEs and HBP signaling pathways in relation to the pathophysiological formation of ROS and pro-inflammatory processes on the functional dysregulation of mitochondrial bioenergetics.

## Introduction

**Fig. 1:**
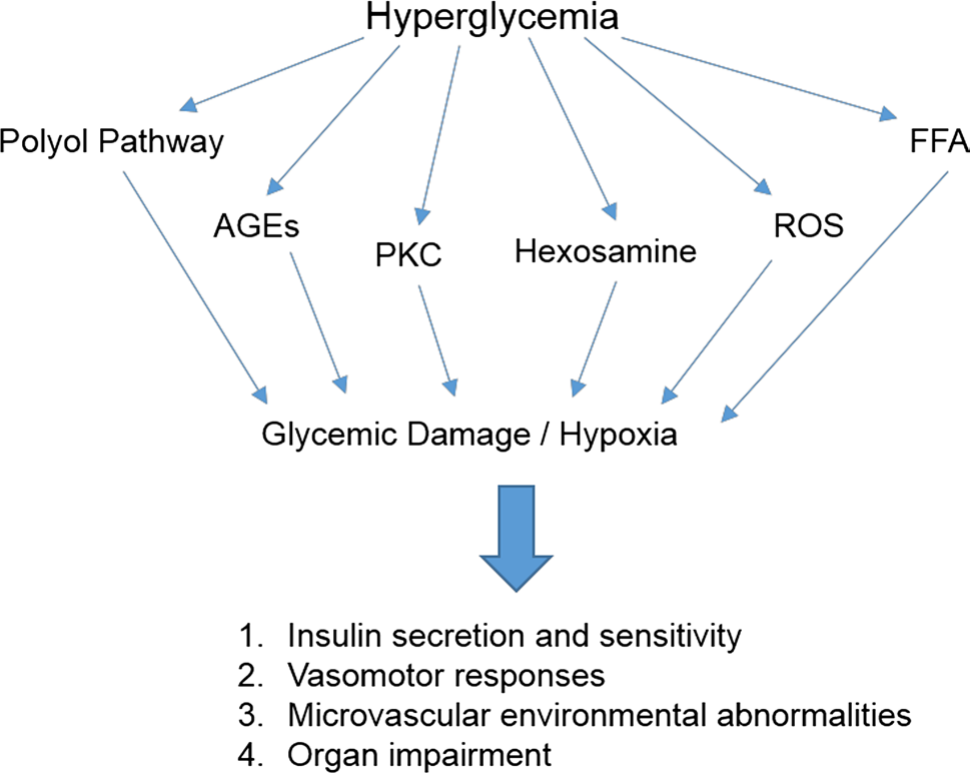
Multiple signaling pathways underlying hyperglycemic cellular damage. As diagrammed, diabetic cellular complications may arise from prolonged exposure to high glucose levels via the intracellular formation of advanced glycation end-products (AGEs), the cognate receptor for advanced glycation end-products (RAGE), and activation of the hexosamine biosynthetic pathway (HBP). Additional signaling mechanisms involved in the induction of hyperglycemia-induced diabetic complications include aberrant phosphorylation events selectively mediated by protein kinase C isoforms and increased flux through the aldose reductase or polyol pathway. A unifying mechanism of diabetic pathophysiology involves hyperglycemia-driven mitochondrial dysfunction and the production of high levels of ROS in the form of superoxide

The severity of untreated or refractory diabetes mellitus has been functionally linked to elevated concentrations of free plasma glucose, clinically defined as hyperglycemia. The pathophysiological presentations of prolonged hyperglycemia may be operationally characterized within insulin-dependent and insulin-independent, type 1 and type 2, diabetic phenotypes, respectively. Accordingly, a relatively broad spectrum of long-term hyperglycemia-associated cellular and metabolic insults has observed in diverse peripheral organ systems and central nervous tissues [1]. Mechanistically, the biomedical literature has focused on the elucidation of key cellular signaling systems that are significantly altered in patients presenting with diabetes-associated chronic hyperglycemia. For example, in both type 1 and type 2 diabetic patients, macro- and microvascular complications may arise from prolonged exposure to high glucose levels via the intracellular formation of advanced glycation end-products (AGEs), which enhance coordinate expression of the cognate receptor for advanced glycation end-products (RAGE) [2]. Chronic hyperglycemia has been functionally linked to aberrant signaling processes mediated by selective enzymes of the hexosamine biosynthetic pathway (HBP), thereby promoting posttranslational modification of key cellular regulatory enzymes and membrane proteins [3]. Additional hypotheses have emerged on the underlying mechanisms of hyperglycemic-induced diabetic complications, including altered expression and signaling by protein kinase C isoforms [4] and increased flux through the aldose reductase pathway [5]. An overriding or unifying mechanism of diabetic pathophysiology may involve hyperglycemia-driven mitochondrial tricarboxylic acid (TCA) cycle dysregulation leading to respiratory complex III dysfunction and the production of high levels of reactive oxygen species (ROS) in the form of superoxide [6]. Presently, we coordinate parallel and convergent published studies evaluating the effects of hyperglycemia on AGEs/RAGEs, and HBP expression in relation to the pathophysiological formation of ROS, into a working hypothesis centering on dysregulated mitochondrial bioenergetics and oxidative stress (Fig. 1).

## Hyperglycemia, AGE formation, and reactive oxygen species

Non-diabetic and diabetic cells by their nature experience significant differences in glucose metabolism and adenosine 5′-triphosphate (ATP) production. In normal cells, initial processing of glucose to triose phosphate intermediates takes place within the cytosolic glycolytic pathway, proceeds with decarboxylation of pyruvate to form acetyl-coenzyme A (Ac-CoA) to fuel the mitochondrial TCA cycle, and terminates with transport of reducing equivalents by membrane-associated respiratory complexes I–IV [7]. The intra-mitochondrial availability of molecular oxygen as the ultimate electron acceptor drives the evolutionarily fashioned chemiosmotic production of ATP as a high-efficiency biological process [8] catalyzed by F1Fo ATPase complexes [9].

Diverse molecular forms of AGEs arise non-enzymatically from the well-characterized Maillard reaction involving a reducing sugar and a primary amino group via Amadori rearrangement intermediate products or secondary addition to Schiff base condensation products [10]. As an example, the prominent AGE glucosepane mediates sustained damage to the extracellular matrix in diabetic tissues, thereby contributing to accelerated sclerotic injury in arteries, kidneys, and other organ systems [11].

In preclinical models of diabetic vascular damage, cultured vascular endothelial cells exposed to hyperglycemic glucose concentrations produce enhanced levels of superoxide, which is functionally linked to increased formation of AGEs and expression of their cognate RAGEs [12]. Because activated vascular endothelial cells release pro-inflammatory cytokines and adhesion molecules, the debilitating increases in oxidative stress mediated by AGEs and RAGEs are also associated with microvascular retinal, glomerular, and nerve lesions in experimental diabetic animals. Importantly, inhibitors of AGEs formation, mitochondrial complex II, or un-couplers of oxidative phosphorylation, were observed to significantly reduce the effects of hyperglycemia on ROS production and cellular damage [12]. Subsequent work linked mitochondrial superoxide formation as a debilitating ROS species facilitating hyperglycemia-associated cellular damage, an effect that was reversed by overexpression of manganese superoxide dismutase (MnSOD) [13]. Combined in vitro and in vivo studies confirmed the functional role of cytosolic ROS in the generation of mitochondrial superoxide at the level of complex I via sustained production of NADH [14]. Interestingly, pharmacologic inhibition of AGE-RAGE-induced mitochondrial permeability transition markedly abrogates the production of mitochondrial superoxide, thereby confirming the pivotal role of AGE-RAGE-induced cytosolic ROS production in the development and progression of diabetic nephron pathologies [14].

Pro-inflammatory mechanisms of action mediated by RAGEs, following stimulation by AGEs, have centered on enhanced expression of the key transcription factor nuclear factor-kappa B (NF-kB) and its targeted genes [15]. Subsequent debilitating cellular processes involve activated monocytes and increased endothelial permeability to macromolecules via inhibition of constitutive NO and enhancement of ROS production and release [15]. Interestingly, RAGE is a member of the immunoglobulin superfamily of cell surface receptors differentially expressed by diverse cell types. Previous work has demonstrated that the promoter region of the RAGE-encoding gene contained three putative NF-kB-like binding sites, thereby linking transcriptional activation and enhanced cellular expression of RAGE to the mediation of severe pro-inflammatory processes [16]. Subsequent work suggests that AGEs themselves are capable of activating RAGE gene expression via NF-kB-mediated processes, resulting in the exacerbation of diabetic microvascular damage [17]. Interestingly, the epigenetic signature of the promotor region of the NF-kB subunit gene RelA/p65/NF-kB3 from peripheral blood mononuclear cells isolated from patients with type 2 diabetes was functionally associated with enhanced transcription of pro-oxidant/inflammatory genes and subsequent vascular damage [18]. The epigenetically mediated upregulation of RelA/p65/NF-kB3 was subsequently linked to the deleterious effects of increased plasma levels of intercellular cell adhesion molecule-1 (ICAM-1) and monocyte chemoattractant protein-1. Similar effects were observed in cultures of human aortic endothelial cells incubated in the presence of hyperglycemic concentrations of glucose [19]. In these studies, the hyperglycemia-induced upregulation of prolyl-isomerase (Pin1) gene expression was functionally associated with nuclear translocation of RelA/p65/NF-kB3 and subsequent enhanced ICAM-1 production. As a validating measure, selective deletion of the RelA/p65/NF-kB3 gene in the livers of transgenic was functionally associated with improved insulin sensitivity [20]. In contrast, overexpression of RelA/p65/NF-kB3 gene activity was associated with enhanced energy expenditure and diminished adipose tissue growth, thereby suggesting that NF-kB-mediated inflammatory processes may have preemptive effects on insulin resistance by eliminating lipid accumulation by adipose tissues [21].

Finally, cell surface expression of RAGE by mast cells has been functionally linked to AGE-mediated apoptotic mechanisms [22]. In an in vitro model, knockdown of mast cell RAGE expression was observed to markedly inhibit AGE-induced apoptosis by blocking mitochondrial Ca(2+) overload and superoxide release. Thus, AGE-induced mast cell apoptosis may contribute to debilitating pro-inflammatory conditions associated with hyperglycemic stress [22].

In summary, a wide array of investigational compounds have been evaluated for their inhibitory activities against diabetes-associated AGE production and/or cellular RAGE expression. For example, aminoguanidine inhibits intramolecular lysyl-arginine cross-linking involved in glucosepane and other AGE formation [11]. Blockade of RAGE-mediated signal transduction has been proposed as a potentially valuable therapeutic strategy for the prevention of hyperglycemia-associated cellular and vascular damage [23]. Notably, a genetically engineered soluble form of RAGE, designed as an AGE-targeted surrogate, inhibits the development of micro- and macrovascular complications arising from chronic diabetic conditions [24].

## Reactive oxygen species, hexosamine biosynthetic signaling pathway, and aberrant mitochondrial function

Dysregulated mitochondrial function has been functionally linked to the etiology and persistence of major metabolic, metastatic, and neurodegenerative disorders [25–32]. Within the HBP, enzymatic formation of O-β-glycosidic linkages of β-N-acetylglucosamine (GlcNAc) to serine and threonine side chains represents a novel posttranslational protein modification of key signaling enzymes and membrane proteins that appears to present strong physiological antagonism to normative signaling processes involving protein phosphorylation [3]. O-GlcNAcylation of proteins appears to be is a dynamic regulatory process mediated by two HBP signaling enzymes O-GlcNAc transferase (OGT) and O-GlcNAcase (OGA), respectively [33]. Accordingly, these regulated enzyme activities are proposed to determine temporally defined intramolecular concentrations of modified signaling proteins via competing processes of addition and removal of O-linked GlcNAc residues [33]. Hyperglycemia-driven posttranslational O-GlcNAc modification of major signaling proteins involved in normative glucose and lipid metabolism has been documented in the biomedical literature, as discussed below [3].

Hyperglycemia-mediated enhancement of mitochondrial superoxide production in vascular endothelial cells is functionally linked to the shunting of glucose into the HBP with resultant long-term activation of pro-inflammatory signaling processes [6]. A key biochemical study has presented empirical evidence supporting the role of hyperglycemia-induced mitochondrial superoxide production as a strong activator of the HBP via inhibition of glyceraldehyde-3-phosphate dehydrogenase (GAPDH) activity within the terminal stages of the cytosolic glycolytic pathway in cultured bovine endothelial cells [34]. Operationally, the functional diversion of the upstream hexose phosphate intermediate fructose-6-phosphate from glycolytic metabolism to the essential substrate N-acetylglucosamine (GlcNAc) by the rate-limiting enzyme glutamine: fructose-6-phosphate aminotransferase (GFAT1) [3], has been proposed as a determining factor in the hyperglycemia-mediated activation of the HBP [34]. The molecular sequelae of aberrant HBP-mediated signaling processes have been linked to enhanced O-GlcNAcylation of transcription factors that subsequently promote expression of genes such as transforming growth factor beta 1 (TGF-B_1_) and plasminogen activator inhibitor-1 (PAI-1) that have been previously established as contributing factors to the pathogenesis of diabetic tissue damage [34]. Finally, in these same studies, decreased GAPDH activity and enhanced HBP activity were reversed by prior addition of an electron transport complex II inhibitor, an un-coupler of oxidative phosphorylation, a MnSOD mimetic, or addition of azaserine, an inhibitor of the rate-limiting enzyme in the HBP, thereby providing essential controls [34].

**Fig. 2:**
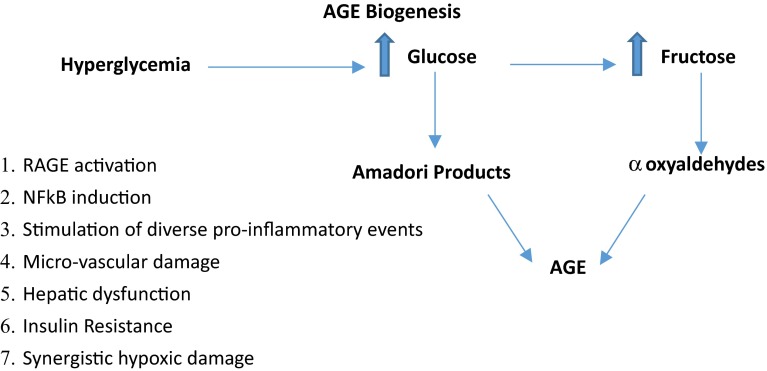
Hyperglycemia-induced enhancement and biological consequences of AGEs production. Advanced glycation end-products (AGEs) are formed from oxidation and derivatization of glucose and fructose. Elevated levels of cellular AGEs are functionally linked to induction of their cognate receptor for advanced glycation end-products (RAGE). The deleterious biological amplification of AGEs/RAGE actions are described in the text

The putative role of altered HBP signaling and selective O-GlcNAcylation of mitochondrial proteins has been explored in a preclinical model of diabetic hyperglycemia utilizing cultured cardiac myocytes [35]. Exposure of cultured cells to hyperglycemic conditions resulted in enhanced O-GlcNAcylation of protein subunits of respiratory complexes I, III, and IV, intimately associated with normative cellular bioenergetics and ATP production. These observations were consistent with the demonstration of diminished cellular ATP levels and mitochondrial Ca(2+) loading [35]. Importantly, increased expression of OGA reduced O-GlcNAc modification of respiratory complex protein subunits functionally linked to enhanced activities of complex I, III, and restoration of normal cellular ATP concentrations. It was concluded that hyperglycemia-driven O-GlcNAcylation of selective mitochondrial proteins is functionally linked to impaired mitochondrial function in diabetic cardiac myocytes. A recent study has demonstrated that perturbations of normative HBP-mediated cycling of O-GlcNAc-linked regulatory and functional mitochondrial proteins profoundly affect cellular bioenergetics as well as mitochondrial morphologies [8]. Utilizing preclinical models of both OGT- and OGA-overexpressing cells, significant diminutions of mitochondrial proteins functionally involved in electron transport processes, oxidative phosphorylation, and the TCA cycle were observed. Interestingly, both cellular respiration/O_2_ consumption and glycolysis were reduced in OGT/OGA-overexpressing cells (Fig. 2).

## Hypoxia and hyperglycemia

The etiology and persistence of major metabolic disorders afflicting diverse human populations are functionally associated with a pathophysiological coupling of systemic pro-inflammatory processes and tissue hypoxia, indicating that ischemic/hypoxic perturbations in oxygen delivery represent significant physiological challenges to the overall viability of multiple organ systems. Reciprocal triggering of multiple ischemic/hypoxic and pro-inflammatory events, if not corrected, will promote pathophysiological processes leading to a deleterious cascade of bio-senescent cellular and molecular signaling pathways, which converge to markedly impair mitochondrial bioenergetics and requisite ATP production.

In light of the above, reciprocal pathophysiological states of hyperglycemia and hypoxia are proposed to induce significant comorbidities in human metabolic diseases. We have recently provided critical discussion on the significance of early hypoxic events on long-term alteration of normative mitochondrial processes in relation to the emergence of pathological states [27, 28, 36–38]. As an example, careful examination of the pathogenicity of diabetic foot ulceration, characterized by poor wound healing, will yield mechanistic links between hypoxic and hyperglycemic conditions and chronic mitochondrial dysfunction [39]. In this regard, increased hypoxic conditions leading to impaired wound healing in diabetic foot ulcerations are functionally associated with impaired hypoxia-inducible factor-1 (HIF-1) expression, an established key regulatory factor in cellular O_2_ homeostasis that mediates the adaptive cellular responses to hypoxic challenges. Furthermore, HIF-1 signaling has been demonstrated to be downregulated in diabetes due to hyperglycemia-induced HIF-1α destabilization linked to functional inhibition [39]. In sum, hyperglycemic-induced “hypoxia” fits into a broadly based pathophysiological scheme with convergent debilitating effects on mitochondrial function. Interestingly, whereas certain cell types have the ability to revert to earlier evolutionary phenotype as an adaptive strategy to hypoxic conditions, these integrative multicellular processes may not be attainable over extended periods of time [28]. To date, advanced mitochondrial targetted therapies have not been forthcoming to address the severe hyperglycemia in concert with chronic hypoxia present in patients afflicted with type 2 diabetes.

## Dietary considerations

The primacy of glucose derived from photosynthesis as an existential source of chemical energy across plant and animal phyla is universally accepted as a core principle in the life sciences. In mammalian cells, initial processing of glucose to triose phosphate intermediates takes place within the cytosolic glycolytic pathway and terminates with the temporal transport of reducing equivalents derived from pyruvate metabolism by membrane-associated respiratory complexes in the mitochondrial matrix. The intra-mitochondrial availability of molecular oxygen as the ultimate electron acceptor drives the evolutionarily fashioned chemiosmotic production of ATP as a high-efficiency biological process. The mechanistic evolutionary bases of diabetes have demonstrated the profound alteration of normative mitochondrial function, notably deregulated respiratory processes leading to the initiation of hypoxia-induced cellular events.

Dietary interventions to counteract debilitating pro-inflammatory and oxidative stress-related cellular events induced by chronic hyperglycemia have received widespread attention in the biomedical literature. Notably, supplementation with omega3-polyunsaturated fatty acids (Ω3-PUFAs) has been proposed as an adjuvant dietary strategy to reduce oxidative stress and lipid peroxidation in obese and diabetic patient populations [40–42]. Accordingly, in rodent models of obesity with or without comorbid diabetes, dietary enrichment with Ω3-PUFAs was observed to reduce triglyceride concentrations, lipid peroxidation levels, and concentrations of AGEs in the livers of treated rats [43]. Dietary supplementation of phospholipid enriched in the Ω3-PUFA eicosapentaenoic acid was observed to partially restore insulin sensitivity and reduce hepatic steatosis in concert with a reduction of pro-inflammatory cytokines in obese [44] or transgenic diabetic mouse models [45]. Furthermore, dietary enrichment with fish containing diverse mixtures of Ω3-PUFAs was observed to promote similar restorative effects on mitochondrial bioenergetics from skeletal muscle of obese/diabetic rodents [46, 47]. Finally, in a relatively recent Japanese clinical study, a reduction of circulating levels of Ω3-PUFAs was functionally associated with higher insulin resistance in cohorts of type 2 diabetic patients [48].

In contrast to the widely reported ameliorative effects of dietary supplementation with Ω3-PUFAs, several lines of investigation have registered concern over the potential pro-inflammatory properties of PUFA metabolites, notably certain molecular species of oxylipins [40, 42, 49]. Oxylipins formed via the action of 12-lipoxygenase on PUFAs to produce pro-inflammatory oxygenated lipid intermediates have been observed to mediate debilitating effects on normative pancreatic β-cell function [49, 50]. Furthermore, oxylipins derived from omega6- polyunsaturated fatty acids (Ω6-PUFAs) in comparison with those derived from Ω3-PUFAs appear to promote a higher degree of cellular damage in obesity-related comorbidities that include insulin resistance, adipose tissue inflammation [42], and non-alcoholic fatty liver disease [40, 41]. In sum, long-term nutritional supplementation with selective PUFAs should be approached with a significant degree of caution with regard to modulation of hepatic and white adipose PUFA content and the potential development of pro-inflammatory processes linked to insulin resistance and hepatic dysfunction.

## Conclusions

Prolonged periods of hyperglycemia mediate major disruptions of normative mitochondrial functions, resulting in chronic exacerbations of pathological conditions affecting many cellular and organ systems. As reviewed, the damaging micro- and macrovascular cellular effects of hyperglycemia are driven by mitochondrial ROS production linked to activation of parallel, but functionally convergent, AGE/RAGE and HBP signaling pathways. In this regard, we speculate that subtle alterations of metabolic homeostasis and its associated pathological abnormalities may have arisen as a result of the evolutionary pressure of unbridled photosynthetic activities within the biosphere with resultant high O_2_ production and abundant levels of glucose which can be consumed by both plants and animals. Furthermore, with the exponentially expanded biological capture of solar energy in the chemical form of reduced carbon molecular species, the evolutionarily fashioned liberation of stored energy linked to reciprocal formation of cellular ATP via the mitochondrial electron transport system was engineered over the course of 1–2 billion years. In the last 5000 years, organismic handling of excessive energy demands has become more pronounced with the advent of modern agriculture. As cognitively driven higher organisms, humans process abundant sources of glucose into “focused comfort-reward” foods, thereby generating an even greater dependence on carbohydrate energy metabolism with potentially dire metabolic consequences [51].
